# Upgrading Sustainable Polyurethane Foam Based on Greener Polyols: Succinic-Based Polyol and Mannich-Based Polyol

**DOI:** 10.3390/ma13143170

**Published:** 2020-07-16

**Authors:** Ferdinando de Luca Bossa, Letizia Verdolotti, Vincenzo Russo, Pietro Campaner, Andrea Minigher, Giuseppe Cesare Lama, Laura Boggioni, Riccardo Tesser, Marino Lavorgna

**Affiliations:** 1 Institute of Polymers, Composite and Biomaterials, National Research Council, P.le Enrico Fermi 1, Portici, 80055 Naples, Italy; fe.delucabossa@gmail.com (F.d.L.B.); giuseppe.lama@ipcb.cnr.it (G.C.L.); marino.lavorgna@cnr.it (M.L.); 2 Department of Chemical Sciences, University of Naples Federico II, Via Cintia 4, 80126 Naples, Italy; v.russo@unina.it (V.R.); riccardo.tesser@unina.it (R.T.); 3 AEP Polymers Srl, Basovizza, 34149 Trieste, Italy; pietro.campaner@aeppolymers.com (P.C.); andrea.minigher@aeppolymers.com (A.M.); 4 Institute for Chemical Science and Technologies, CNR, V. Corti 12, 20133 Milano, Italy; laura.boggioni@cnr.it

**Keywords:** sustainability, rigid polyurethane foams, succinic-based polyol, Mannich-based polyol, 1,4-butanediol, succinic acid monomers

## Abstract

It is well known that the traditional synthetic polymers, such as Polyurethane foams, require raw materials that are not fully sustainable and are based on oil-feedstocks. For this reason, renewable resources such as biomass, polysaccharides and proteins are still recognized as one of the most promising approaches for substituting oil-based raw materials (mainly polyols). However, polyurethanes from renewable sources exhibit poor physical and functional performances. For this reason, the best technological solution is the production of polyurethane materials obtained through a partial replacement of the oil-based polyurethane precursors. This approach enables a good balance between the need to improve the sustainability of the polymer and the need to achieve suitable performances, to fulfill the technological requirements for specific applications. In this paper, a succinic-based polyol sample (obtained from biomass source) was synthesized, characterized and blended with cardanol-based polyol (Mannich-based polyol) to produce sustainable rigid polyurethane foams in which the oil-based polyol is totally replaced. A suitable amount of catalysts and surfactant, water as blowing reagent and poly-methylene diphenyl di-isocyanate as isocyanate source were used for the polyurethane synthesis. The resulting foams were characterized by means of infrared spectroscopy (FTIR) to control the cross-linking reactions, scanning electron microscopy (SEM) to evaluate the morphological structure and thermal gravimetric analysis (TGA) and thermal conductivity to evaluate thermal degradation behavior and thermal insulation properties.

## 1. Introduction

Sustainable resources such as biomasses can be used as a valuable and inexhaustible feedstock to produce several chemicals, as they are naturally renewable. In this context, the production of polyols from biomasses has been widely investigated worldwide in both academic institutions and industries, since they represent, along with polyisocyanates, the main components for the manufacturing of sustainable polyurethanes [1,2,3,4,5]. These polyols, obtained from biomass through fine chemical processes, represent an environmentally friendly alternative to the continuous depletion of fossil resources [6,7]. Their diffusion may contribute to the reduction of the price of polyurethane foams due to the large availability of resources and making the resulting foams more sustainable. However, the research in this field and the optimization of the chemistry to obtain polyols are still at an early stage, with wide margins to make the overall sustainability strategy competitive with the traditional oil-based products. In fact, it was proven that renewable polyols are still not able to support the production of high-performance polyurethane foams and huge efforts in this field are needed to allow the polyols to become competitive and comparable with petrochemical raw materials, in terms of having the right functionality and molecular weight, as well as good final properties of the foam [6,7,8,9].

Actually, the feasibility of using bio-based polyols to produce polyurethane foams (PUs) has been strongly investigated due to the wide application fields accessible for foamed products such as biomedical, construction, aerospace, etc. Several chemical approaches were proposed to synthesize suitable polyols from biomass. Generally, the extraction of building block molecules (levulininc acid, succinic acid, fatty acid, etc.) from biomass sources is involved, producing the polyhydroxyls via transesterification, amidation, oxirane ring opening, or oxypropylation reactions [10,11,12]. Those functionalization processes allow one to obtain a wide variety of polyhydroxyls based on oil crops, suggesting interesting intermediates for designing polyurethanes with novel macromolecular architectures [10,11,12].

Among the building blocks from biomass [13], succinic acid [14,15,16] is an example of a bio-molecule that can be obtained only via a bio-based pathway, since an identical fossil-based counterpart (e.g., 1,4-butanediol, levulinic acid, polylactic acid polyhydroxyalkanoate) is not reported in the literature. Furthermore, it represents a good bio-based platform/building block to produce a lot of chemicals and polymeric precursors (such as polyols). Actually, as reported by the European report on the market of bio-based chemicals [13], differently from the other bio-based molecules, succinic acid, as it can be found in several biomass sources, is widely present in the “green molecules market”, being estimated in the global market at around 47.5 kt/a in 2014 and constantly growing. For this reason, researches addressed to verify and optimize the use of this “unit” as an alternative “raw material” for polymers production (such as polyurethanes) with thermal, mechanical and chemical–physical properties comparable to the synthetics ones, are strongly encouraged because it remains an issue. In this respect, using succinic acid (along with 1,4-butanediol) as a chemical to synthesize succinic-based polyols is yet to be explored.

**Scheme 1:**
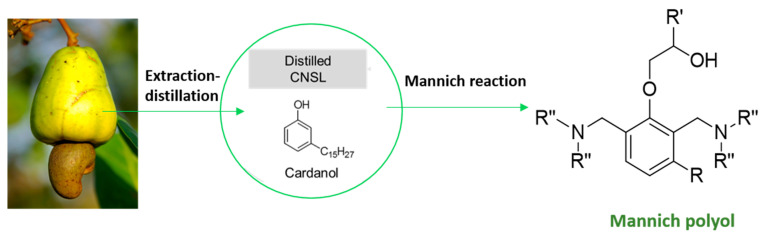
Schematic representation of the synthesis of the Mannich-based polyol. CNSL: Cashew Nut Shell Liquid.

Among bio-based polyols, such as Mannich-based polyols [17,18,19,20] derived from a bio-source, or Cashew Nut Shell Liquid (CNSL) [17,18], due to their chemical structure (phenolic ring and a tertiary amine; see Scheme 1) they can be used for the synthesis of bio-based polyurethane. For instance, cardanol (building block) is recovered from CNSL through several chemical processes (extraction, distillation) and then, subsequently, through a condensation reaction, a Mannich polyol is synthesized.

The high aromaticity brings valuable intrinsic benefits to foams such as thermal, fire and chemical resistance, while the aliphatic part leads to the improvement of water resistance and wetting properties. Furthermore, due to the presence of a high content of tertiary nitrogen, Mannich polyols are more reactive than conventional ones. For this reason, they are used in combination with other polyols, both polyesters and polyethers, in rigid polyurethane formulations [17,18,20].

However, since in reality polyurethane foams produced starting from bio-based polyol represent a good alternative, having characteristics comparable or improved, with respect to traditional petroleum-based polymers, the replacement of fossil-based polyols in polyurethanes is still receiving significant attention [21,22].

In this paper, sustainable polyols were synthesized starting from a bio-based building block, succinic acid (and 1,4-butanediol), obtained by a fermentation process of biomass, as extensively described in Stanzione et al. [14].

In a previous work [14], a succinic-based polyol (bS) was blended (at two different percentages 50 and 80 wt%) with a conventional succinic-based polyester polyol (code: Biosuccinium Polyol-FF1) to synthesize bio-based flexible polyurethane foams. The resulting cellular morphology highlighted the presence of a partial open cellular structure with small pores and a tendency for incomplete cell opening by increasing the amount of bS, until 80 wt%, with respect to the total amount of polyol [14]. However, the authors concluded that the produced foams showed behavior comparable to the conventional flexible polyurethane foams.

Differently to the previous paper [14], herein the authors evaluated the possibility of using succinic-based polyol (bS) (blended with a sustainable Mannich polyol (bM)) to produce rigid polyurethane foams with good thermal stability and thermal insulation properties.

The bS was produced and characterized from the chemical and thermal points of view, and subsequently three different bS/bM ratio (namely 30/70, 50/50 and 70/30 wt%) were used to formulate the innovative bio-based rigid polyurethane foams.

Finally, the chemical, morphological and thermal properties of produced samples were thoughtfully assessed.

## 2. Materials and Methods

As reported and detailed in a previous paper by Stanzione et al. [14], succinic acid (SA) was produced starting from Arundo Donax biomass, through a pilot plant in which hydrolysis and fermentation experiments, along with several ultrafiltration processes, without using solvent [15]. The succinic acid as it is was used as raw material to synthetize 1,4-butanediol, via succinic acid hydrogenolysis, catalyzed by Ru/C heterogeneous catalyst as discussed by Turco et al. [16].

Mannich polyol (Cardolite code: GX-9104, namely bM), was kindly provided by AEP Polymers. Catalysts, used to control the polymerization and blowing reactions (CH_3_COOK and Niax PM40), and silicone surfactant to stabilize cells (L5111) were provided by Momentive (Termoli, Italy). Poly-methylene diphenyl isocyanate (P-MDI, Suprasec 8025, NCO = 30.5%) was kindly provided by Huntsman (Ternate, Italy). Distilled water was used as a blowing reagent to produce CO_2_ blowing agent. No flame retardant was used due to the high thermal stability of Mannich polyol (GX-9104).

### 2.1. Procedure for the Synthesis of Succinic-Based Polyol

**Figure 1:**
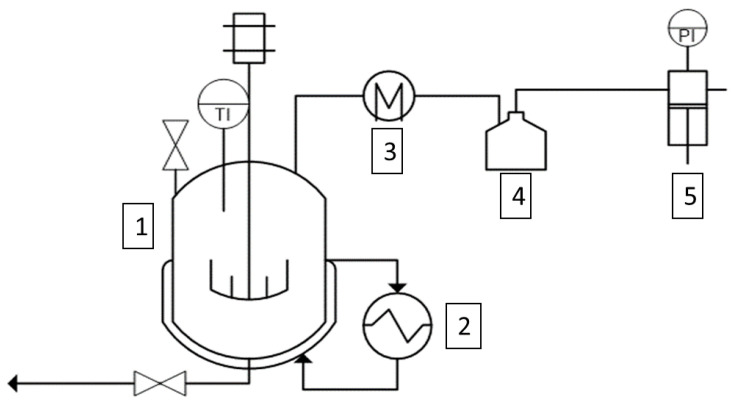
Sketch of reactor set-up of succinic-based (bS) polyol. 1: Reactor; 2: Heat exchanger device; 3: Condenser; 4: Collecting tank; 5: Vacuum pump.

The synthesis of succinic-based polyol [8,14] was conducted in a 0.25 L batch reactor (see device sketch in Figure 1). The mixture of succinic acid/1,4-butanediol at a given molar ratio is loaded into the reactor, where the temperature is adjusted by using a heating oil bath, regulated by an automatic heating cuff: temperature is measured using a thermocouple placed in the reactor vessel. A distillation system is connected at the top of the reactor to allow the removal or either water or diol formed as the reaction proceeds. This equipment works at low pressure, using a vacuum pump, where an intermediate refrigerated trap allows any damage to the vacuum system. The vacuum was finely regulated by adjusting a dedicated valve.

The reaction consists of two different steps:

(i) Esterification reaction: loading of the reactants followed by heating the system at the desired temperature value at atmospheric pressure, 1 h reaction time:

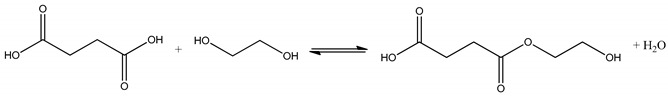
(1)

(ii) Polycondensation reaction: adding TNB catalyst, stirring for 1 h under high vacuum.

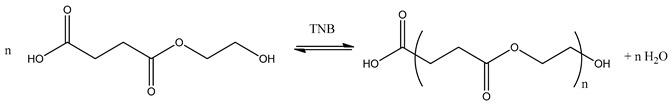
(2)

The resulting product (namely “bS”) was quickly quenched and collected. The experimental conditions adopted in this synthesis step are reported in Table 1. The molar ratio between succinic acid and 1,4-butanediol was fixed at 1:1, as it was demonstrated to be the best bio-polyol from our previous work [14], but differently to the previous paper the amount of catalyst (TNB) was reduced from 1 wt% to 0.26 wt% (percentage referred to SA).

### 2.2. Polyurethane Foam Preparations

Polyurethane foams were produced by fixing the ratio of NCO/OH equal to 1.3. The foam samples were prepared through two steps procedure: (1) bS polyol (30, 50 and 70 wt%, namely PS3, PS5 and PS7) was melted at 103 °C and then added, at 100 °C and under stirring, to the bM polyol previously formulated with a suitable amount of catalysts, surfactant and blowing reagent. (2) Subsequently, P-MDI was quickly added under stirring to the formulated blend polyols. No evaporation was observed during these steps. The resulting mixture was poured in a prismatic mold at 70 °C for 24 h. Due to the high aromaticity of bM (12.4%), any flame retardant was used in the formulation. The details of polyurethane foams formulations are summarized in Table 2.

**Figure 2:**
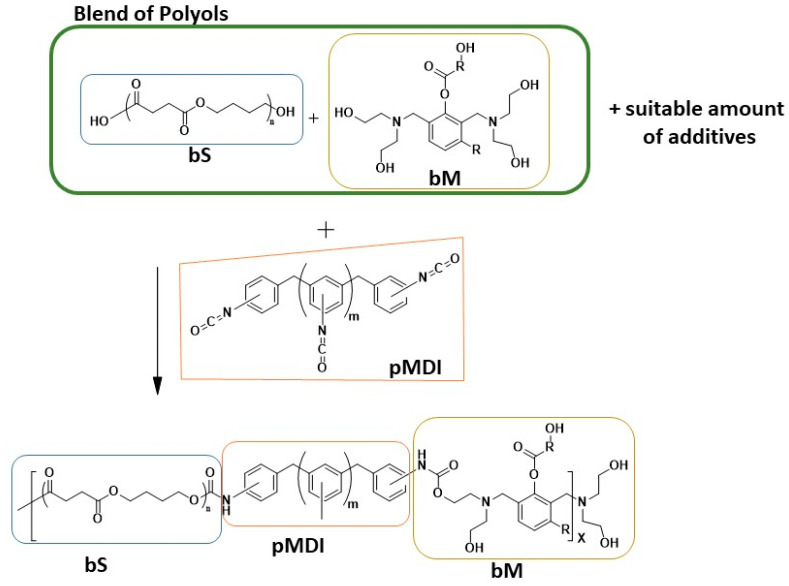
Sketch of general chemical structure based on bS and Mannich polyol (bM) polyols.

The produced foams (see in Figure 2 the scheme of general chemical structure of foam produced starting bio-based polyols) were characterized from the chemical, morphological and thermal points of view.

A polyurethane foam with 100 wt% of bM (namely PS0) was also prepared for proper comparison.

### 2.3. Characterizations of Polyols

Succinic acid conversion was determined through acid titration, to measure the residual acidic groups of the synthesized polyols. An aliquot of 0.1 g of the sample was dissolved in 10 g of THF. A standard methanolic solution of NaOH (0.1 M) was used as a titrating agent, while phenolphthalein was chosen as the indicator. The residual percentage of carboxylic group was calculated by dividing the moles of carboxylic acid groups at the equivalent point ($n_{\mathrm{COOH}}$) by the moles of the acidic groups obtained by titrating the prepared reaction mixture ($n_{\mathrm{COOH}}^{0}$) (see Equation (3)), (procedure reported in detail in [14]).

(3) $$R_{\mathrm{COOH}}=\frac{n_{\mathrm{COOH}}^{0}-n_{\mathrm{COOH}}}{n_{\mathrm{COOH}}^{0}}\;100$$

The hydroxyl number (I_OH_) of produced bS was also evaluated according to ASTM D4274-05, E Method.

To evaluate the glass transition temperature and the melting point of both bM and produced polyol (bS, which at room temperature was a solid wax), differential scanning calorimetry (DSC, using a DSCQ1000-TA Instrument (New Castle, DE, USA) with a scan rate of 10 °C/min from −50 to 250 °C) and thermal gravimetric analysis (by using TGAQ500-TA Instruments (New Castle, DE, USA), with a scan rate of 10 °C/min and in the Temperature range 25 °C to 800 °C, in nitrogen atmosphere) were used to assess the thermal degradation temperatures.

The measurements of glass transition (@DSC analysis), melting point (@DSC analysis), as well as thermal degradation temperatures (@TGA analysis), were carried out by using TA software [23,24].

To analyze the chemical structure of produced polyol, infrared spectroscopy (FTIR) spectra on bS and bM were recorded at room temperature through FTIR spectrometer (model Frontier Dual Ranger, PerkinElmer, Waltham, MA, USA) in attenuated total reflectance (ATR) mode from 400–4000 cm^−1^. ATR spectra were collected on the samples as made. Spectra were recorded at 4 cm^−1^ resolutions, and the reported results are the average of 64 scans [25].

Nuclear magnetic resonance spectroscopy (^1^H-NMR) analysis for bS were recorded in CDCl_3_ and Bruker spectrometer (Bruker Italia srl, Milan, Italy) operating at 400MHz. Tetramethylsilane (TMS, Chemical shift: δ = 0.00 ppm) was used as internal standard [14].

### 2.4. Characterizations of Foams

To evaluate the chemical structure of produced foams produced foams, FTIR investigation was provided. The FTIR analysis was recorded at room temperature through an FTIR spectrometer (model Frontier Dual Ranger, PerkinElmer, Waltham, MA, USA) in attenuated total reflectance (ATR) mode from 400–4000 cm^−1^. ATR spectra were collected on the surface the foam samples. Spectra were recorded at 4 cm^−1^ resolutions, and the reported results are the average of 64 scans [3,14,24].

Scanning electron microscopy (SEM) investigations (FEI Quanta 200 FEG scanning electron microscope-ESEM, Eindhoven, The Netherlands) were used to observe the morphological microstructure, mainly to evaluate the closed or open cellular structure [26,27] of produced samples. Diameters were determined by analyzing SEM images via ImageJ software (public domain Java image processing program, software open source). Each cell was considered as an ellipse, having two semi-axes. The average value obtained from these two values was the sought diameter.

Thermal gravimetric analysis (TGA) analysis, using a TA Instrument (New Castle, DE, USA), was used to evaluate the thermal degradation properties of produced foams by using a scan rate equal to 10 °C/min from room temperature to 1000 °C, in inert atmosphere [24].

To assess the thermal insulating capability of polyurethane foams, thermal conductivity characterization was evaluated at room pressure and temperature by using the modified transient plane source (MTPS) technique on a C-Therm TCi thermal conductivity analyzer (New Brunswick, Canada). The C-Therm analyzer is comprised of a one-sided interfacial heat sensor (with a 17 mm of diameter) that applies a momentary constant heat source to the sample with a measurement pulse between 1 to 3 s. Cylindrical foams (with diameter 6 cm and thickness equal to 3 cm) were put on the sensor.

#### 2.4.1. Sustainability Index of the Developed Foams

To evaluate the degree of sustainability of the produced samples, the authors introduced a new factor, arbitrarily defined as the “Sustainability Index (*S.I.*)”, which gives a measure of the sustainability of the examined system, i.e., the weight percentage of the mass derived from sustainable components with respect to the total mass of the foam. The “*S.I.*” is calculated with the following arbitrary Equation (4)*:*

(4) S.I.=100×(S.P.[g]/T.W.[g])

where “*S.P*.” is the “Sustainable Part” which is represented by the sustainable polyols, for example extracted by biomasses from several natural sources (vegetable oils, marine, fish processing, wastes agricultural wastes, secondary raw materials, etc.), whereas *T.W.* is the total weight of the foams.

## 3. Results

### 3.1. Polyols Characterizations

The synthesized bio-based polyhydroxyls were characterized through FTIR and ^1^HNMR analysis to assess their chemical structure.

**Figure 3:**
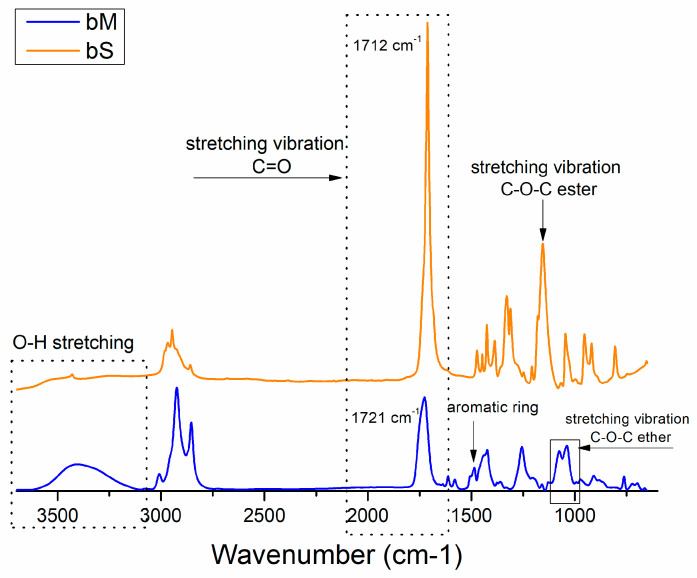
Infrared spectroscopy (FTIR) spectra of bio-based polyols: bS and bM.

The FTIR analysis was conducted on bM polyol and synthesized bS polyol, and the spectra are reported in Figure 3. By observing the FTIR spectra, it was observed for the bS spectrum that the main characteristic peaks related to the OH stretching vibration around 3300 cm^−1^, the carbonyl absorption peak at 1711 cm^−1^ (higher than C=O of bM due to its higher quantity in the polymeric structure) and the stretching vibration C-O-C at 1153 cm^−1^, both ascribed to the polyester polyol [5,7,8,14]. Conversely, bM polyol shows the OH and C=O absorption peaks (see the spectrum in Figure 3), the double peaks around 1100–1000 cm^−1^ belong to the C-O-C ether groups of Mannich polyol and the C-N and aromatic vibration peaks at 1254 cm^−1^ and 1490 cm^−1^ wavenumber, respectively [18,28,29,30].

**Figure 4:**
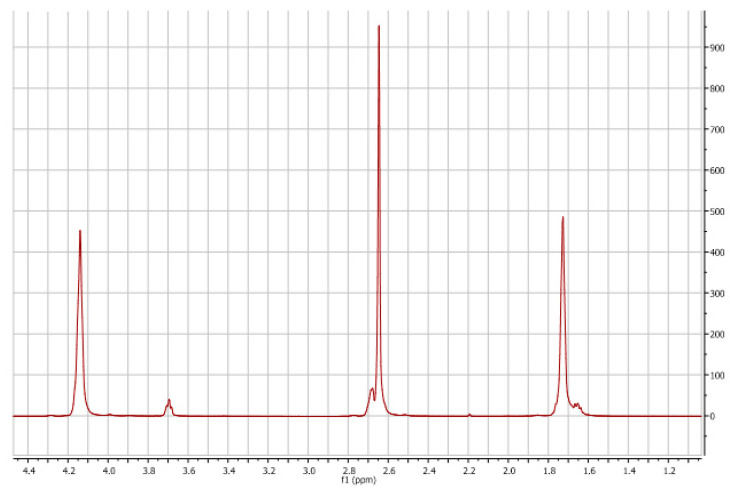
^1^H-NMR spectrum of synthesized bS polyol.

The ^1^H-NMR analysis was conducted on the synthesized bS polyol to gain further information about the chemical nature of the prepared products. The results are given in Figure 4.

As observed, in the spectrum at around *δ* = 1.65–1.68 ppm a singlet correlating to the (C**H**_2_)CH_2_OH protons is present, while at *δ* = 2.6 ppm the CH_2_COOCH_2_ group can be observed. The formation of polyhydroxyl described in the reaction scheme is given by the singlet at *δ* = 4.18 ppm, corresponding to (C**H**_2_)_2_OCO (ii) [14].

The succinic acid conversion was determined by characterizing the bS polyol in terms of the amount of acidic groups. Furthermore, the *I_OH_* number was also assessed and the value is reported in Table 3 along with the content of H_2_O and the weight average molar mass (determined in Stanzione et al. [14]). The results reported in Table 3 on bS show a total esterification as the succinic acid conversion degree (*R*_COOH_) approaches the stoichiometric value (*R*_COOH:_ 93%).

**Figure 5:**
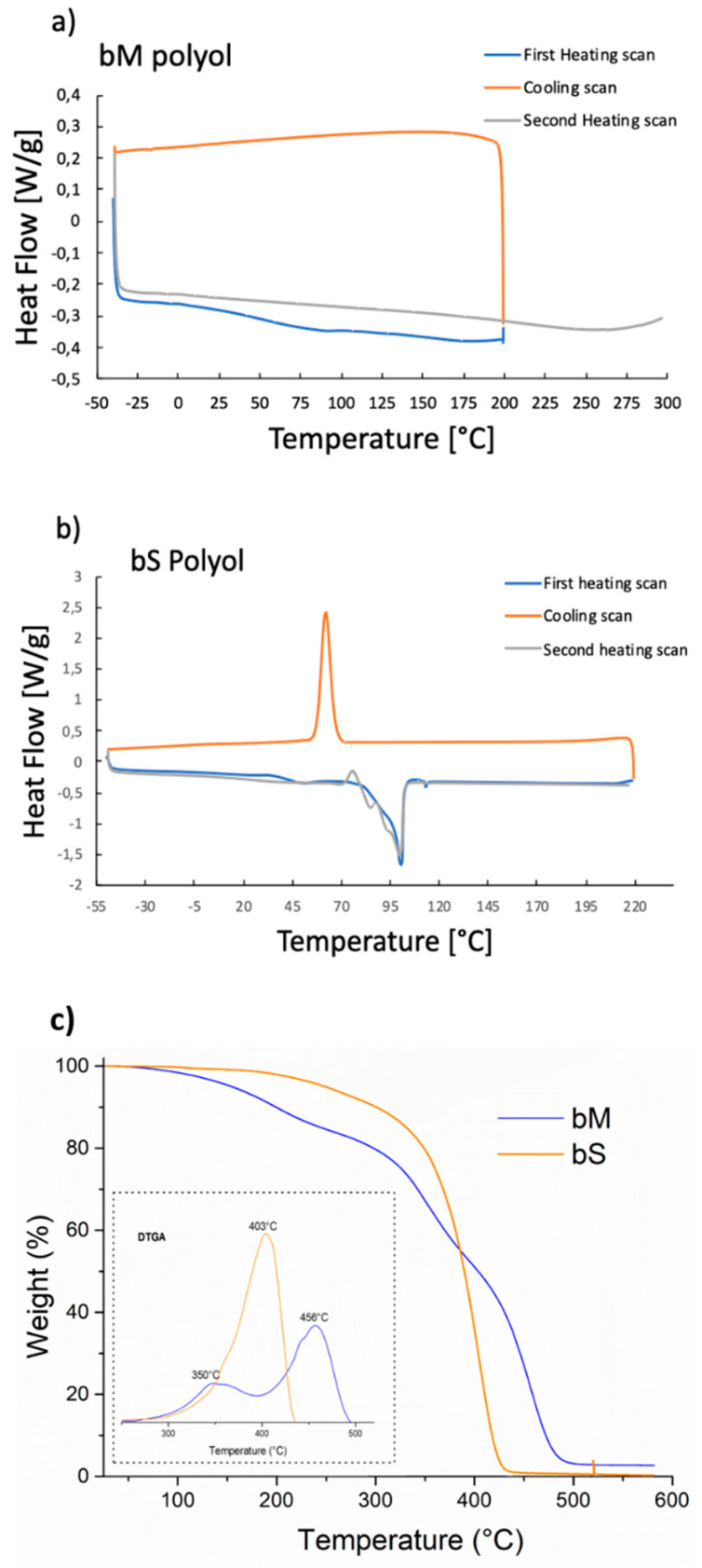
(**a**) Differential scanning calorimetry (DSC) of bM polyol. (**b**) DSC of bS polyol. (**c**) Thermal gravimetric analysis (TGA) and derivative TGA (DTGA) of bM and bS polyols.

Due to the fact that the polyurethane foam synthesis occurred at high temperatures (>100 °C), a thermal characterization of polyols (bS and bM) through DSC and TGA analysis was conducted to assess the thermal phenomena and mainly the value of the degradation temperatures. In Figure 5 the DSC (Figure 5a,b) and the TGA graphs are reported.

The DSC curve related to the bM polyol (Figure 5a) in the first scan shows an inflection point due to a transition phase corresponding to the glass transition temperature (T_g_) [31,32] around 75 °C. No other phenomena were recorded. In the second scan, along with the T_g_ at 75 °C, an endothermic peak with a maximum at 250 °C was present, which could be correlated to the evaporation of aliphatic or aromatic hydrocarbon molecules with low molecular weight present within the bM polyol, as also hypothesized by Lopes et al. [31,32]. In the case of the bS DSC thermogram (Figure 5b), a semi-crystalline behavior was observed due to the presence of either a first- or second-order thermodynamic transitions. A melting point at 103 °C (T_m_) ascribed to the crystalline region (first-order transition) and the inflection point around 55 °C (T_g_) related to the chains in amorphous phase (second-order transition) were detected [23,33]. This result is in accordance with the behavior of succinic-based polyesters reported by Bikiars et al. [33].

From TGA analysis (Figure 5c), bS polyols revealed a significant degradation step that starts around 300 °C (maximum of the derivative weight loss, T_m1_ around 303 °C) with a weight loss equal to 90 wt%, attributed to the decomposition of polyols in its volatile molecules such as CO_2_, alcohols, etc. Conversely, the bM polyol revealed two main degradation steps which occur in the range of 300–400 °C (with a maximum of the derivative weight loss, T_m1_, around 356 °C and a weight loss equal to 40 wt%) and 400–500 °C (with a maximum of the derivative weight loss, T_m2_, around 452 °C and a weight loss equal to 56 wt%). In these ranges the two decompositions were ascribed to the breaking of links related to the linear hydrocarbon chain present in the bM, while at higher temperature to the breaking of aromatic constituents of Mannich polyol. The higher thermal stability of bM, due to the presence of the aromatic species, induces a profound effect on the stability of the derived polyurethane foams [17].

### 3.2. Polyurethane Foams Characterizations

**Figure 6:**
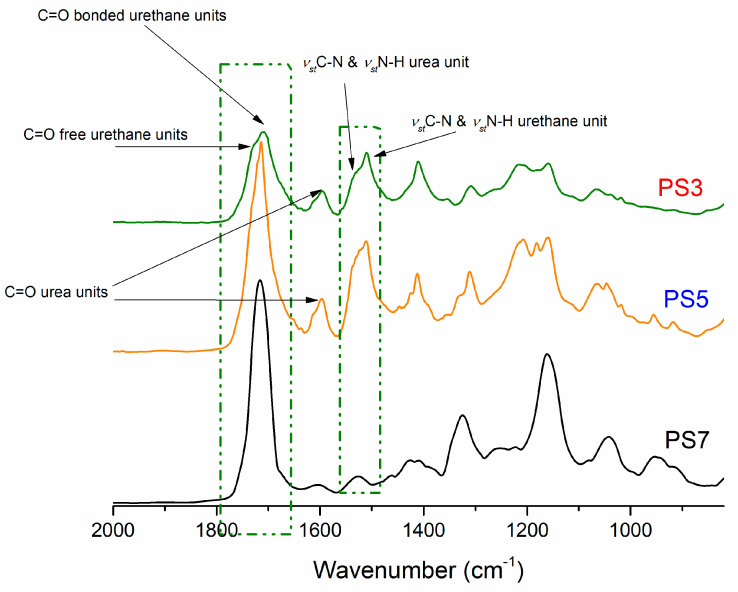
FTIR spectra of polyurethane foams: PS3, PS5 and PS7.

Figure 6 reports the FTIR spectra related to the PS3, PS5 and PS7 samples (the FTIR spectrum of PS0 is reported in de Luca Bossa et al. [7]), with the focus in the main characteristic region of urethane groups (2000–800 cm^−1^). The absence of the signal related to the asymmetric stretching of free NCO group (~2270 cm^−1^, data not reported for brevity), in all the spectra, confirms the occurrence of the polyurethane reaction as well described elsewhere [4,7,9,14]. The peak at 1705 cm^−1^ assigned to the characteristic stretching vibration of C=O hydrogen-bonded urethane units was observed for PS3. Alongside with this an additional peak, present as a shoulder, at 1728 cm^−1^, assigned to the stretching vibration of C=O (free carbonyl group) of the urethane units, was also detected [34]. As reported by several authors [35,36], the free C=O groups arise from the hydrogen interactions between hard segments (urethane linkages) and soft segments (polyol linkages, i.e., ester groups). Instead, H-bonded C=O arise from the interactions between the hard segments (inter-urethane bonded hydrogen) [35]. The PS5 and PS7 samples highlighted a C=O absorption peak at 1711 cm^−1^ and 1716 cm^−1^, respectively, due to the overlapping of both C=O hydrogen-bonded urethane units and free C=O (the last one at the higher quantity). The results showed that by increasing bS content, the intensity of C=O increasing (due to the higher amount of C=O in the polyester bS polyol) and the structure of the polyurethane shows more C=O free groups and thus a lower amount of “hard domains” in the polymeric phases. This means that the macromolecular structure of PS5 and PS7 was less constrained [36] as compared with the structure of the PS3 system. Furthermore, in the PS3 and PS5 spectra, vibration peaks correlating to the urea linkages were also observed (see the spectra in Figure 6) [7,35].

**Figure 7:**
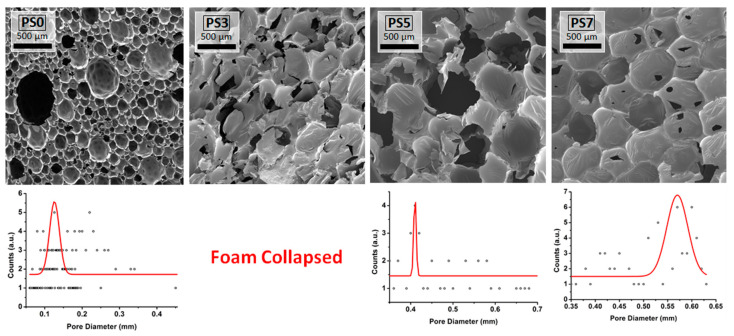
Scanning electron microscopy (SEM) micrographs of produced PS0, PS3, PS5 and PS7.

**Figure 8:**
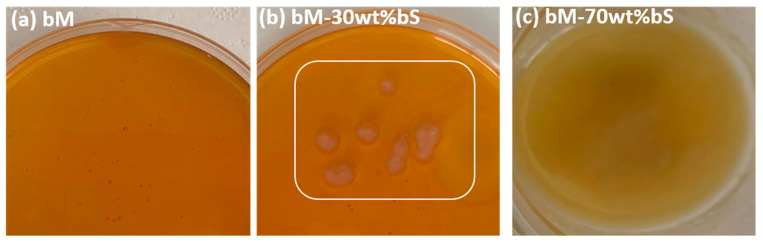
Mixture of (**a**) Pristine bM polyol, (**b**) bM-30wt%bS and (**c**) bM-70wt%bS polyols.

The morphology of polyurethane foams and the corresponding surface cell diameter distribution are reported in Figure 7**.** As observed, the pristine PS0 exhibited a somewhat homogeneous cell distribution (diameters ranging from 100 to 450 µm) with a closed cells structure [7,26,37] typical of a rigid polyurethane foam [6,7,24,26]. By adding the bS, a change in morphology was observed. The addition of a low amount of bS (30 wt% with respect to bM) induced a collapse of the foam structure, which can be tentatively ascribed to a poor miscibility (see the picture in Figure 8b) that occurred during the addition of bS into bM polyol at 103 °C; the miscibility of bS in bM is very low and at this concentration there is a clear component separation, with droplets of bS clearly identified in the bM matrix (Figure 8b), as also reported for other polyester polyols/CNSL-derived polyols blends [29,30]. Conversely, by increasing the amount of bS, the miscibility improved as highlighted in Figure 8c (the two polyol components started to be somewhat miscible at 50/50 wt/wt), and a foamed structure with a partially closed cell structure and a larger sized surface cells distribution was observed for PS5 (see the distribution for PS5 in Figure 7). At high concentration of bS polyol (PS7), the Mannich polyol was completely miscible into the bS matrix, and the resulting polyurethane foams exhibited a homogeneous structure with a closed cells structure morphology and a narrow cell diameter distribution centered around 500 µm.

**Figure 9:**
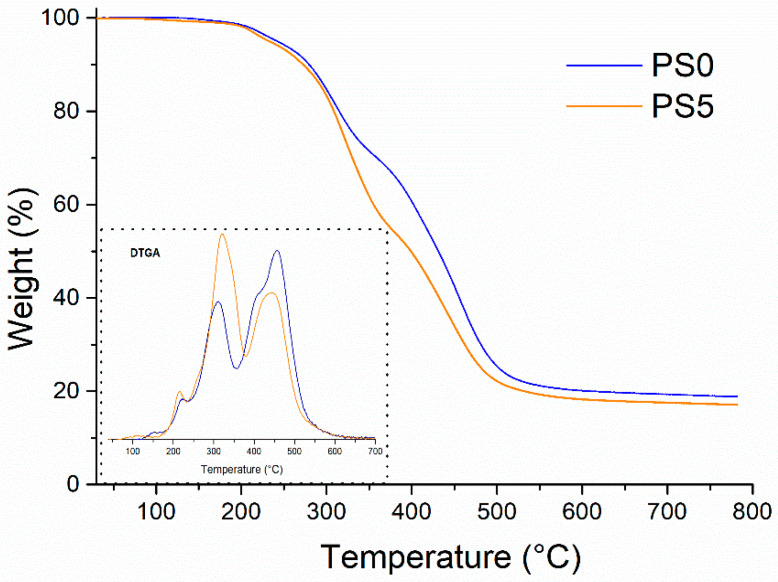
TGA and DTGA of selected produced bio-based foams, PS0 and PS5.

Table 4 reports the thermal properties of produced foams from TGA analysis (selected thermograms are reported in Figure 9) and thermal conductivity investigation. Generally, polyurethane foams show three degradation processes, as described elsewhere [24]. Low levels of weight loss (around 5–7 wt% for all samples), correlating to the evaporation of small molecules (such as water or species with low molecular weight), can be observed in the 120–240 °C range (maximum of the weight loss derivative is namely T_max1_). The second degradation step, which occurs in the range of 230–350 °C (T_max2_), is correlated to the break/rupture of urethane bonds, with the formation of polyol segments and high amounts of isocyanate which became more complex products, with the evolution of volatile compounds. At this step weight losses equal to 29 wt%, 41 wt% and 49 wt% for PS0, PS5 and PS7 respectively were recorded. Finally, the aforementioned complex products degrade at higher temperatures (>350 °C, T_max3_), the third degradation step. The last degradation moved towards higher temperature for the foams with the highest amount of Mannich-polyol due to its higher thermal stability with respect to the bS polyol.

In fact, as observed in Table 4**,** the maximum decomposition temperatures and the char amount (residue @800 °C) of the foams decreased with the addition of bS and the consequent reduction of the bM polyol, which is well known to present a high thermal stability ascribed to its high aromaticity [19,38]. The high aromaticity leads to a high yield of char during the thermal treatment process of the resulting foams and can confer to the material an inherent fire resistance [38]. Because the char layer acts as a protective/barrier layer (thermal insulator and mass transport barrier) during the burning process, it prevents the propagation of flame, holds back the flammable gases and isolates the heat from unburned material.

Thermal conductivity values for the foams herein described are typical of polyurethane-based thermal insulation panels obtained by using water as blowing agent. In particular, for PS0, the λ value was 0.031 W/m·K, for PS5 0.035 W/m·K (due to a much more open morphological cell structure), while for the PS7 sample a reduction in thermal conductivity (0.029 W/m·K), with respect to the other foams, was recorded. This can be correlated to its closed cells structure and the decreasing of density.

As extensively described by several authors [7,37,39,40], the thermal transfer (λ_i_) in a foamed material is described by the combination of four pathways (heat transfer through solid phase/matrix, λ_s_, heat transfer by gas phase present in the porous structure λ_g_, thermal radiation through the cell walls, λ_r_, and the thermal convention within the cells, λ_c_ that is negligible for the foam with cell size smaller than 4 mm [7,37,40,41], as reported in the following Equation (5):

λ_i_ = λ_s_ + λ_g_ + λ_r_ + λ_c_ [W/(m·K)] (5)

It clear that the reduction of λ_i_ is derived by the reduction of one of the aforementioned terms. As describe by Gibson et al. [42], the contribution of λ_s_ is low for the PU foams (~10%) due to the low thermal conductivity of polymeric phase (that is 0.25 W/(m·K)) and due to the fact that the solid phase occupies a small volumetric fraction of the total volume of the foam. The λ_g_ contribution, which is equal to the product between the thermal conductivity of the gas (CO_2_ for our foams) and volumetric fraction of the gas foam, changes with relative density (ρ*/*ρ_s_*, where ρ* is the foam density and *ρ_s_* is the density of the not expanded polymer [42]) of the foam and of course to the closed cell percentage. Finally, the radiation factor [42,43,44,45], λ_r_ [46], depends on the cell dimensions, the wall thickness (for instance foams with a high number of cells with small size dimensions transfer less heat by radiation than foams with a few big cells), along with the higher closed cell amount. For these reasons, in our case the closed cells structure and the reduction of foam density (ρ*) can be responsible of the reduction of the terms λ_g_, λ_s_ and λ_r_, and consequently of λ_i_.

These outcomes confirm that the bS can be successfully used at high concentrations to prepare rigid polyurethane foams with a closed cell structure by blending it with the sustainable Mannich polyol at 70 wt%. Furthermore, the presence of Mannich polyol, due to its chemical structure, permitted obtaining a foam with a good thermal degradation stability, without the addition of any flame retardant in the formulation.

#### Sustainability Index of the Developed Foams

The Sustainability Index, “*S.I.*”, an arbitrary factor, was introduced in this paper in order to assess the sustainability of a given sample. It was determined considering the amount of eco-sustainable components used for the production of the selected samples, with respect to the total weight, as explained in the Section 2.4.1.

Several papers [47] report the production of polyurethane foams by using those naturally derived as a partial substitute of the petroleum-derived polyol.

The authors evaluate the “*S.I.*” for the Polyurethane foams produced herein and it was compared with the “*S.I.*” of those obtained by the recent literature [22,48,49,50,51,52,53,54]. In particular, polyurethane foams with densities below 100 kg/m^3^ and with the maximum amount of sustainable quantity were considered among those discussed in the selected literature.

**Figure 10:**
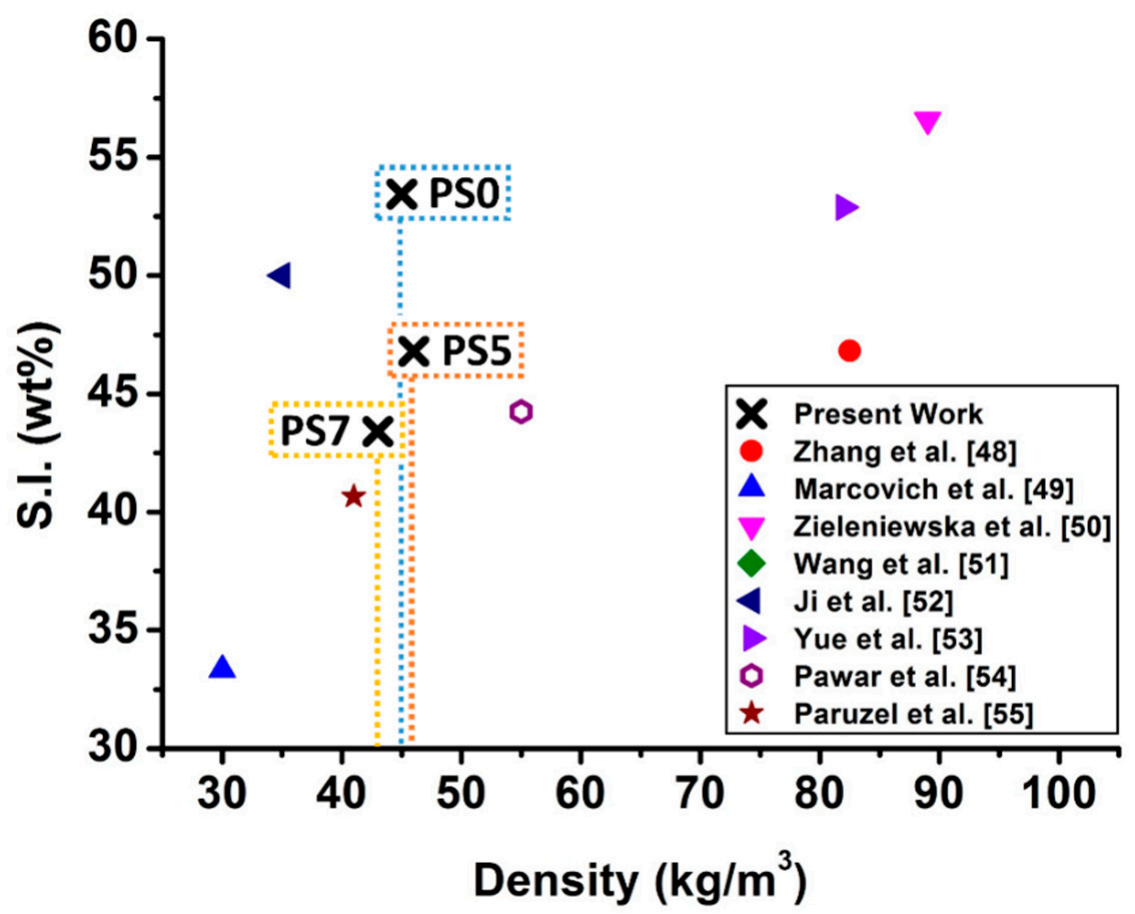
Sustainability Index of PS0, PS5, PS7 and of polyurethane foams from selected papers.

In Figure 10 the results are displayed on the graph in which the “*S.I*.” is reported as function of density. It is possible to observe that, despite the fact that our systems were realized by using a high amount of sustainable reagents, the densities were kept at low values, confirming the high sustainability level with respect to the Polyurethane foams obtained from the literature at comparable density.

## 4. Conclusions

In the previous article, the authors verified the potential application of three types of succinic-based polyhydroxyls as precursors to synthesize flexible polyurethane foams. Differently from this, in the present research the authors have produced a polyol using a lower amount of catalysts, and evaluated the possibility of using succinic-based polyol as a precursor for the preparation of rigid foams. In particular, herein sustainable rigid polyurethane foams were successfully synthesized by using a blend of Mannich- and succinic-based polyols (at three different wt/wt ratios) produced starting from Cardanol and biomass sources, respectively. Significant differences are recorded between the three foams types, due to the relative miscibility of the two polyols. For instance, the foams with a low concentration of succinic-based polyol (30 wt%) collapse due to the low miscibility with Mannich polyol. However, the foams with a higher amount of succinic-based polyol (70 wt%), thanks to its good miscibility (at this concentration) with Mannich-based polyol, have displayed a closed cell structure, a lower density and consequently a lower thermal conductivity value (0.029 W/(m·K)). Furthermore, the presence of Mannich-based polyol provides a high thermal stability, even if there is no flame retardant, which is higher at higher Mannich-polyol concentrations. This means that the produced systems could be optimal candidates to be used as a thermal insulating foam in the construction sector. Moreover, it has been assessed that the sustainability content, compared with that of polyurethane foams obtained from literature data with the same density, by means of an arbitrary factor, the “Sustainability Index”, is higher.

## Figures and Tables

**Table 1:** Experimental reaction conditions for the synthesis of bS polyol. T (°C): process temperature; SA: succinic acid; B: 1,4-butanediol; xTNB: catalyst weight percent referred to SA.

Sample	T(°C)	xTNB (wt%/SA)	SA/B (mol/mol)
bS	150	0.26	1:1

**Table 2:** Polyurethane foams formulations.

Sample	bM (wt%)	bS (wt%)	P-MDI (wt%)	KAc (wt%) *	PM40 (wt%) *	L5111 (wt%) *	H_2_O (wt%) *
PS0	53.5	-	45	0.27	0.27	0.53	0.53
PS0	30	13	44	0.30	0.30	0.50	0.43
PS5	23.4	23.4	51.2	0.60	0.47	0.47	0.47
PS7	13	30	54	0.52	0.52	0.6	0.43

* wt% with respect to 100 parts of total polyol.

**Table 3:** Characteristics of bS and bM polyols.

Sample	R_COOH_ (%)	I_OH_ (mg KOH/g)	H_2_O Content (wt%)	Mw * (g/mol)	Viscosity (cPs)@25 °C	T_g_ (°C)	T_m_ (°C)
**bS**	93	430	0.01	5292	-	54.6	103
**bM**	-	245	≤0.1	-	3500–7500	75	-

* Weight average molar mass (Mw) [16].

**Table 4:** Thermal properties of PS0, PS3, PS5 and PS7 foams.

Sample	Foam Density (kg/m^3^)	T_max1_ (°C)	T_max2_ (°C)	T_max3_ (°C)	Char (wt%)	λ (W/m·K)
PS0	45	222	318	416	19	0.031
PS3	-	-	-	-	-	-
PS5	46	215	315	415	16	0.035
PS7	43	206	310	400	14	0.029
